# Oppression and internalized oppression as an emerging theme in accessing healthcare: findings from a qualitative study assessing first-language related barriers among the Kurds in Turkey

**DOI:** 10.1186/s12939-022-01824-z

**Published:** 2023-01-07

**Authors:** Tevfik Bayram, Sibel Sakarya

**Affiliations:** 1 School of Public Health, University of Montreal, 7101 Park Ave, Montreal, QC H3N 1X9 Canada; 2 Department of Public Health, School of Medicine, Koç University, Topkapı, Koç Üniversitesi Hastanesi, Davutpaşa Cd. No:4, Zeytinburnu, 34010 Istanbul, Turkey

**Keywords:** Access to healthcare, Language, Kurdish, Oppression, Internalized oppression, Kurds, Turkey

## Abstract

**Background:**

Language has been well documented to be a key determinant of accessing healthcare. Most of the literature about language barrier in accessing healthcare is in the context of miscommunication. However, it is critical to consider the historical and political contexts and power dynamics underlying actions. The literature in this matter is short. In this paper we aimed to find out how first-language affects access to healthcare for people who do not speak the official language, with a particular focus on language oppression.

**Methods:**

We conducted this qualitative study based on patient-reported experiences of the Kurds in Turkey, which is a century-long oppressed population. We conducted 12 in-depth interviews (all ethnically Kurdish, non-Turkish speaking) in Şırnak, Turkey, in 2018–2019 using maximum variation strategy. We used Levesque’s ‘Patient-Centred Access to Healthcare’ framework which addresses *individual* and *structural* dimensions to access.

**Results:**

We found that Kurds who do not speak the official language face multiple first-language related barriers in accessing healthcare. Poor access to health information, poor patient-provider relationship, delay in seeking health care, dependence on others in accessing healthcare, low adherence to treatments, dissatisfaction with services, and inability to follow health rights were main issues. As an unusual outcome, we discovered that the barrier processes in accessing healthcare are particularly complicated in the context of oppression and its internalization. Internalized oppression, as we found in our study, impairs access to healthcare with creating a sense of reluctance to seek healthcare, and impairs their individual and collective agency to struggle for change.

**Conclusions:**

A human-rights-based top-down policy shift, and a bottom-up community empowerment approach is needed. At the system level, official recognition of oppressed populations, acknowledgement of the determinants of their health; and incorporating their language in official capacities (particularly education and healthcare) is crucial. Interventions should include raising awareness among relevant professions and stakeholders that internalized oppression is an issue in accessing healthcare to be considered. Given that internalized oppression can be in other forms than language or ethnicity, future research aimed at examining other aspects of access to healthcare should pay a special attention to internalized oppression.

**Supplementary Information:**

The online version contains supplementary material available at 10.1186/s12939-022-01824-z.

## Background

Language has been well documented to be a key determinant of accessing healthcare (HC) [1, 2]. Growing evidence shows that language barriers interfere with access to HC at patient, provider, and system level [1, 2]. It is associated with under- and over-use of certain services [3, 4]; dissatisfaction of patients and providers [1, 2]; miscommunication between patients and providers [1, 2], higher costs at patient and system level [1, 2]; and poor health outcomes [1–4]. Most of the literature about this issue has addressed language in the context of miscommunication [1, 2]. However, language is not only a mean of communication, it signals the speaker’s group affiliation and consequently functions as a reference to social position [5, 6]. Thus, it grants advantages and/or disadvantages in social interactions and leads to inter-personal and/or structural racism [6, 7]. As a result, it inequitably affect HC use of certain populations, such as migrants, ethnic minorities and native populations [1, 4, 8, 9]. Therefore, it is critical to consider the historical and political contexts and power dynamics when studying language and health [10, 11]. The literature in this matter is short. In this paper we addressed language barrier in accessing healthcare, based on patient-reported experiences of the Kurds in Turkey, with a particular focus on language oppression.

The Kurds in Turkey is estimated to be around 20% of the country’s whole population which corresponds to around 16 millions [12] The majority of the Kurds (66%) live in the Eastern part of the country [13]. For around a century, the Kurdish language has been systematically suppressed as part of the aim to construct a homogenous Turkish-speaking nation-state [14, 15]. During this period, the Kurdish language has been rendered invisible, inferiorized, and sometimes criminalized through severe regulations and prohibitions [14–17]. Thus, Turkish has become the lingua franca for all citizens to use in public and official capacities [15]. Despite the loosening of suppressive policies in the last three decades, with such as introduction of private Kurdish courses in 2003, and elective Kurdish classes in middle schools in 2012 [15], Kurdish is still excluded from official capacities, including education and healthcare.

It is unknown what percentage of the Kurds in Turkey do not speak Turkish. Nonetheless, since Turkish is taught through formal education to Kurds, the level of education can be a proxy measure of Turkish speakers. In 2011, a study found 17% of the Kurdish population are illiterate and can be considered non-Turkish speaking; and 9% are literate with no formal education [13] and can be considered having limited comprehension of Turkish (the rate of illiteracy among the Turkish population is 4% according to the same study) [13]. Current studies also show that around 30% of Kurds have primary-school or lower education level; and around 70% speak Turkish in their daily life [18, 19]. Resulting in approximately a quarter of the Kurdish population (around 4 million people), having a limited comprehension of Turkish. Data in Turkey shows that people who do not speak Turkish as their first language have significantly poorer access to healthcare [3]. However, no study has been conducted to understand how it interferes with access. In this regard, based on the experience of the Kurds in Turkey, we strived to find out the answer(s) for the greater question “how does first-language affect access to healthcare for people who do not speak the official language?” with a particular focus on language oppression.

### Theoretical background

Access to healthcare is widely accepted as a key factor affecting health outcomes, and is central in the performance of healthcare systems worldwide [20, 21]. Although etymologically access is defined as a way of approaching, reaching, or entering a place [22]; *access to health care* is a more complex notion. Levesque et al. describe access to health care as “the opportunity to identify HC needs, to seek HC services, to reach, to obtain or use HC services and to actually be offered services appropriate to the needs” [21]. In this study, we explored access to healthcare using Levesque et al.’s ‘Patient-Centred Access to Health Care framework’. This framework addresses access to healthcare in 5-steps: perception of needs and desire for care, HC seeking, reaching, utilization, and consequences. The framework is important with its conceptualization of *structural* and *individual* dimensions determining access. The five structural dimensions are: approachability, acceptability, availability, affordability, and appropriateness. The five individual dimensions are: ability to perceive, seek, reach, pay, and engage. In this study, we address both individual and structural dimensions through individuals’ (patients’/users’) perspectives.

Examining access to healthcare through patients’ perspectives is relevant for this study because it addresses aspects of healthcare that are difficult to measure, such as satisfaction, participation, perceptions, and preferences [23–26]. This perspective also provides data on “why” problems occur [24]. Thus, it makes a unique contribution to planning efforts for improving healthcare services based on users’ own needs and expectations [24]. This approach is in line with the World Health Organization’s framework on integrated people-centered health services, which calls for health systems to prioritize people when developing health systems [26].

In this article, in which we examine how first-language affects access to healthcare, we also discuss the concept of oppression and its internalization [11]. Freire defines oppression as: “any situation in which ‘A’ objectively exploits ‘B’ or hinders his and her pursuit of self-affirmation as a responsible person…”. According to Freire, the oppressors’ consciousness desires to “transform everything surrounding it into an object of its domination” [11]. Freire emphasizes how the objectification of the oppressed in society results in the internalization of oppression. The oppressed internalize the image oppressor holds of them, adopt what is prescribed by the oppressor and believe that the punishment, violence, or condemnation they receive is deserved [27]. Oppression is both a state and a process. As a state, oppression creates unequal group access to power and privilege, and as a process, it tries to maintain inequality between groups [28]. Oppression, therefore, results in the differentiation of people into groups (e.g., dominant/dominated, powerful/powerless, superior/inferior, oppressor/oppressed), and group membership determines the degree to which an individual has power or the opportunity and ability to access resources [29].

In this paper, the power dynamics and political/historical contextualization we address are based on ethno-linguistic opression. We are aware that there are other power relations likely to impact access to healthcare, such as those based on gender inequalities, sexual orientation or socio-economic status. However, in our study, the focus is power relations based on ethno-linguistic characteristics.

## Methods

We collected the data through face-to-face in-depth interviews between April 2018 and January 2019 in Şırnak city center and a village 11 km away from the city. The interviews were held in Kurdish by a Kurdish male researcher from a similar background as the participants (first author). We selected 12 participants purposively using the following criteria: being ethnically Kurdish, not speaking Turkish, having a preexisting health condition. Additionally, we used the ‘maximum variation strategy’ to maximize the diversity of factors associated with access to HC, such as gender, age, health status, and distance from HC services (interviewee profiles are given in Table 1). We interviewed people who had a preexisting health condition so that they would need to seek healthcare. We did not recruit participants from health facilities to avoid interviewing those who already accessed HC. We selected some participants through personal contacts, and some using the snowballing method. In the snowballing method we did not accept participants from close relatives and from the same neighborhood to ensure diversity of opinions. Every interview, except one (UW37), provided permission for audio recording. The interviews were held mainly in the interviewee’s houses. We reached saturation with 12 interviews, therefore, we did not hold further interviews. Saturation was decided with no major expansion of codes or categories in the last two interviews.

**Table 1:** Interviewee profiles

Interviewee code	Age (years)	Gender	Health conditions	Place of the interview	Interview duration (minutes)
RW51	51	Woman	Dental problems, previous pregnancies and births	Rural	52
RW49	49	Woman	Goiter, breast cysts, chronic pain	Rural	26
RM62	62	Man	Kidney cysts, chronic pain	Rural	30
RM60	60	Man	Chronic pain	Rural	38
RM67	67	Man	Hypertension, arthritis	Rural	33
RW33	33	Woman	Previous pregnancies and births (pregnant during the interview)	Rural	27
UW66	66	Woman	Heart disease, hypertension, osteoporosis	Urban	27
UM63	63	Man	Myocardial infarction (operated)	Urban	42
UM59	59	Man	Disc hernia (operated twice)	Urban	27
UW67	67	Woman	Hypertension, arthritis	Urban	36
UW37	37	Woman	8 childbirths	Urban	25
UM68	68	Man	A urinary system disease, headache	Urban	37
	Mean: 51.7				Mean: 33.3

Interviewee codes: U represents urban; R represents rural; W represents woman; M represents man: the number after the letters represents the age of the participant. For instance, RW51 refers to a rural woman who is 51 years old

We learned from the key contacts that the main reason for the refusal of male participants was the fear that “something [politically unfavourable]” could happen to them, because participating in an interview in Kurdish was overly-politicized. For women, however, the main reason was gender roles. Because the interviewer was a man, some had to ask for permission from their husband, and some did not agree to be interviewed alone. One interview (UW37) was conducted with the presence of the interviewee’s daughter and neighbor; and one (UW67) with the interviewee’s husband. All the interviews were held by a male researcher, because we couldn’t find a Kurdish-speaking female researcher. We don’t know the actual non-response rate because the key contacts did not keep a record of the people they contacted through the snowballing method and refused to participate. We provided 100 Turkish Liras (≈US$20) incentive for participation.

### Interview guide

We used Levesque’s [21] framework of access to healthcare as a basis for our semi-structured interview guide, and strived to cover all the individual and structural dimensions. Simultaneously, the structure of the interview guide was flexible to permit new topics (codes, categories and themes) that could be raised by the interviewees. For each stage, we asked content mapping and mining questions [30] to obtain a deeper and fuller understanding. To test the applicability of the interview guide, we first conducted two pilot interviews. In these interviews the two researchers made necessary changes in the interview guide.

### Data analysis

We applied a thematic analysis and followed the subsequent hierarchy [30]: transcription, data management, descriptive account, and explanatory accounts. Firstly, a professional who holds a master’s degree in Kurdish Language transcribed the interviews. Then we uploaded the texts to the Atlas.ti program. Afterwards we used a combination of deductive and inductive coding and applied thematic analysis. Deductively, we used Levesque et al.’s framework of access as an analytic structure. We accepted most of the components in the Levesque’s model as a code or category. However, the coding process was flexible to permit new topics beyond Levesque’s model. Therefore, inductively, we allowed emergence of new codes and categories, one of which was internalized oppression. The coding, categorization and the primary analysis was done by TB and the remarkable quotes were translated to Turkish (since SS doesn’t speak Kurdish). In the descriptive accounts (see Results), we identified key elements, refined categories, classified the data; and tried to find patterns of associations within the data. In this part (Results), we used an emic perspective, namely we presented the opinions of the participants from their own perspectives [31]. In the explanatory accounts (see Discussion), we developed explanations for the patterns of associations found in the descriptive accounts. In this part (Discussion), we used an etic perspective, namely we presented the interpretation of the opinions from the perspective of the observers’/researchers’ [31].

We followed the Consolidated criteria for reporting qualitative research (COREQ) [32] guideline in writing this paper.

## Results

**Fig. 1:**
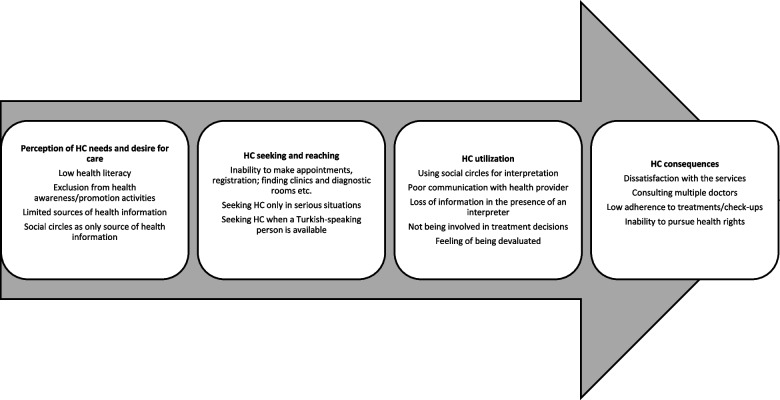
First-language-related barriers in accessing healthcare for the non-Turkish speaking Kurds in Turkey (an adopted version of Levesque et al.’s [21] framework)

We organized our findings around four main stages of access in Levesque’s framework: *perception of needs and desire for care; HC seeking and reaching; HC utilization; and HC consequences*. At each stage, we found that participants pointed to individual and/or structural dimensions related to access to services to varying degrees. As summarized in Fig. 1, we found multiple language-related barriers at each stage. We also found an emerging theme beyond Levesque’s framework – oppression and internalized oppression – as a contextual factor that complicates language-related barriers in access to HC. As such, we inductively applied this new theme to the related stage of Levesque et al.’s framework.

### Perception of needs and desire for care

We found that the participants had limited information about their health conditions and healthcare system, for example: whether they need to see a doctor or not; and when and whom they should visit. They reported their main source of health information as their social circles (mainly relatives, majority of whom also have low education and limited comprehension of Turkish). They stated that they are generally not able to ask questions to their healthcare providers. In cases where they have a Kurdish family physician or nurse, they sometimes visit Family Health Centers to ask questions from them.

*“If I don’t ask someone, I don’t know [what specialist I should visit] and I don’t know who [specialist] is where. (RW51)”. If they [the doctors] are Kurdish… For instance, the one [family physician] here is Kurdish, like you and me, I go and ask him. (RW51)” “Everybody goes to him. Even those who have no problems go and ask something. (RW33)”.*

### Healthcare seeking and reaching

Most of the participants stated that they have never visited a doctor on their own. And those who visited health care services generally did it when their health condition became serious. They described a dependence on a trustworthy Turkish-speaking person (generally a relative) to accompany them. However, they stated that their relatives are not always available, and even if they are available, especially for the participants who have chronic conditions, they don’t feel comfortable calling them regularly.

*“I have never been to a doctor on my own. I haven’t been able to do that (RW49)”. “Once I went on my own, but I came back the way I went (UM68)”.*

*“If they [my kids] are home [I can ask them to accompany me]. But I feel guilty when my son misses a class because of me. I am their mother but still… (UW66)”.*

*“Last year I went to the doctor. He put me in that closed cabinet [MRI machine]. I waited for the results for one year. I waited until my grand-daughter [who is a university student in another city] returned home… If I could do it by myself, I would visit the doctor 1-2 times in a month. (UM68)”.*

We also found that they are not able to make an appointment and again are dependent on a Turkish-speaking person. After the appointment, they still need a person to complete their registration at the health facility and take them to the doctor’s/nurse’s office or diagnostic test rooms.

*“It is very hard. They will ask you to take a blood test or a tomography… If someone is not with you, you will just go back and forth. (RW49)”.*

At this stage, we also found a pattern of behavior that went beyond language and rooted in internalized oppression. In response to the question about the use of emergency services, most of the participants stated that they woudln’t call (or haven’t called) an ambulance, not only because they couldn’t speak Turkish, but because they had a low self-worth for the service.

*“The other day I had excruicating pain in my kidneys. I asked my son to call his uncle to rush me to the hospital (RW51)”.*

*“I don’t speak Turkish, it [ambulance] would never even come to my mind. (RM60).” “And why would I bother an ambulance in the middle of the night. (RM62).” “Why would an ambulance come for me, such a poor guy.(RM67)”.*

### Health care utilization

We found that the communication between patients and healthcare providers is done through an interpreter who is generally a relative and also has a limited comprehension of Turkish. There is a (perceived) loss of information between patient and provider through the interpreter. There are also sensitive issues that are not discussed because of the presence of the interpreter.

*“I go with my daughter. If she is not with me I will never go, even if I die here... (UM68)”. “[The doctor] asks the person [interpreter], he then asks me and I tell him... I am not comfortable with that at all. You don’t know if he [the interpreter] said everything as you explained or not. And sometimes there are things that you can’t say. [RW51].” “For instance you have pain in your private parts, you can’t say it. [Normally] you would say it to your doctor, there is no shame with the doctor, but you are ashamed when your children or your neighbor is there. (RM60).” “I would prefer a person who I don’t know [personally]... (UW37).”*

We found that the participants are not able to build a dialogue with health care providers. The patient-provider relationship is generally a ‘monologue’, rather than a ‘dialogue’. Based on the experience of our participants, it appears that they are almost exclusively ‘receivers’ of transmitted information, rather than being an active part of discussion or treatment decisions. We found that this type of patient-provider relationship is not only related to language, but also internalized oppression. When the participants talked about their experiences with doctors; while with Turkish doctors their general mood was discomfort, shyness, silence, and strict compliance; with Kurdish doctors it was comfort, confidence, chattiness, non-compliance (negotiation) and a sense of humor. Therefore, they preferred health providers who speak Kurdish.

*“When it’s your language, it is like eating on your own. But when it is not your language, it is like being fed with a spoon by someone else” (UM59).*

*“I have no relationship with them [doctors], nothing. (UM68)” “Once he [the doctor] gave me a pill, it was red. After taking two pills I felt dizzy. So I stopped taking it. The next time I went to him I didn’t mention it. (RM67).” “If it was in my own language, I would say everything that was on my mind. I would say hey Mr. or Mrs. Doctor, look I have this and this. (RM60).” “For instance, our family physician is Kurdish, whenever I go he would say ‘hey uncle! Welcome! Is your blood pressure high again?’ I would say yes of course… [laughing] [describing a conversation with a sense of humor] (RM67).*

Another indicator of oppression and its internalization at this stage was the reluctance of Kurdish-speaking health professionals to speak Kurdish with their patients. RW51 mentioned that some health professionals, despite being able to speak Kurdish, don’t speak Kurdish with them.

### Health care consequences

One of the common consequence of the language barrier was that the participants often postpone or cancel their treatments. They also have low adherence to treatmet and regular check-ups (particularly those who have chronic conditions).

*“If I don’t feel very bad I don’t go to doctors (RW49)”.*

*“It would be nice to know what medication [prescribed] is for what problem. I don’t know what it is for and what is inside it. I just take it, even if it would be poisonous. (RW51).” “Sometimes you take a pill, and you feel worse [adverse effects], you don’t know what to do. Sometimes you tell [your doctor], but most of the time you don’t. (RW51)”.*

Another consequence of language barrier was the inability to pursue health rights. Moreover, the participants were using a self-directing tone for not being able to pursue their health rights which indicates internalized oppression. The main pattern of speech was: “I wish I could speak Turkish” rather than “I wish the services were provided in my language”.

*“Once a car hit my daughter, they took her to the hospital. I went there and the police called me. They blamed my daughter. I couldn’t say anything. I was saying [to myself], ah ah, I wish I could speak Turkish! Then you would see who is guilty and who is innocent. But you can’t claim your rights, they do whatever they want. (UM63)”.*

In regard to stigma and discrimination, the majority of the participants stated that they haven’t experienced it. Most of them supported the general idea that *“all doctors want to treat their patients*”. However, some participants said, on some occasions they felt ‘devaluated’ because of not speaking Turkish. Consequently, this has led to the emotions of feeling sorry for themselves, anger or guiltiness. We posit that this perceived feeling of being devaluated is an indicator of internalized oppression.

*“I haven’t directly experienced [stigmatization or discrimination]… but they [doctors] probably think why these people are so illiterate. (RW51).” “Because I don’t speak Turkish, they would consider themselves superior, they would say [internally] ‘look at this, he doesn’t speak Turkish, he can’t even reply to my questions’, and then I would feel sorry for myself. (UM68)”. “I wish I could talk to them on my own. This [not being able to communicate with doctors] makes me angry. (RW49)”. “I hate myself, I want to explode. (UM59).”*

Another language related consequence was when the patients are referred to other cities for their treatments, they lose their social support, and feel more desperate.

*“Cizre [a district in the province] is ours, it’s our language, I can talk with someone, I can see someone who I know of. (RM60).” “In Diyarbakir [a neighboring metropolitan city], we suffered a lot. I would go to the parks [in the area] and cry until our appointment time. (UW66)”.*

## Discussion

In this paper, which aims to find out first-language related barriers in accessing healthcare, we found multiple barriers in each stage (perception of HC needs, HC seeking, reaching, utilization); as previously indicated in the literature [1, 2]. For the *perception of HC needs and desire for care*, access to health information and inadequate health literacy emerged as the main themes [33] Access to health information is an important component of access to healthcare particularly as knowledge to desire for care [33], as well as having more control over decisions about health and well-being [34]. For *HC seeking and reaching*, postponement of seeking healthcare emerged as the main theme. Particularly, patients with chronic diseases were vulnerable to losing their companions/interpreters due to regular need for healthcare which led to postponement. For *HC utilization*, decreased effectiveness of utilization emerged as the main theme. Due to the use of interpreters -particularly unprofessional- loss of information and miscommunication emerged as a significant barrier between patient and provider. As a *consequence*, language barrier was found to be associated with low adherence to treatment and dissatisfaction with services. Most of these findings have been previously discussed in the literature [1–4]. Contrastly, we found that all these processes become overly complicated in the context of political oppression and its internalization. In the following section we will discuss our findings about how oppression and its internalization complicate language-related access to healthcare.

One of the impacts of internalized oppression was the reluctance to seek healthcare. The paramount example of this was when a participant stated that “why would I bother an ambulance”. The participant did not intend to call an ambulance not only because he couldn’t speak Turkish, but because he thought that he was not worthy of the service. Because in his mind the ambulance belonged to a superior structure that when he called for it, he would be a ‘bother’. This form of self-depreciation is a characteristic of the oppressed populations, mentioned by Paulo Freire, which derives from internalization of the opinion the oppressors holds of them [11].

The pattern of internalized oppression was seen in the patient-provider relationship as well. When talking about their experiences with doctors; with a Turkish doctor, the general mood was discomfort, shyness, silence, and strict compliance; with a Kurdish doctor it was comfort, confidence, chattiness, non-compliance (negotiation) and a sense of humor. A similar pattern of internalized oppression was also seen in the form of discrimination. The main form of discrimination was not explicit discrimination but rather the implicit feeling of being devalued by healthcare providers. We believe that not mentioning explicit discrimination might be due to two reasons. First, explicit discrimination might be happening in rare occasions, second, discrimination might not be reported during the interviews because of the perceived consequences of structural discrimination.

The indicators of internalized oppression were also noticeable throughout the interviews, hidden in the participants’ language. The participants were using a self-directing tone in their answers. The main pattern of speech was: “I wish I could speak Turkish” rather than “I wish healthcare services were provided in my language”. This is a characteristic of the oppressed, mentioned by Freire, in which the oppressed are convinced of their own unfitness to the system [11]. However, this undermines the autonomy and dignity of individuals and communities, which in turn destroys their agency and potential for making change [11, 35–38]. Because in their mind, the one should change is not the system, but the oppressed themselves [11]. This is against the basic rule of health promotion: enabling people to increase control over and improve their health [39]. Wishing to speak Turkish can also be an ‘adaptive preference’, namely a preference based on the options available [40]. Since Kurdish is not an available option, it makes the participants wish to learn Turkish out of neccesity. Because, in present circumstances, speaking Turkish could be empowering and help individuals navigate the state bureaucracy while making political demands in favor of bilingualism and provision of public services including education and healthcare in their first language.

Another impact of oppression and its internalization was that it voided commonly used coping strategies against language barriers. One of the main coping strategies for dealing with language barriers is ethnic matching [1, 41]. In other words, people who do not speak the official language attempt to find a healthcare provider who speaks their language. In situations where they can find a match, language barrier becomes less pronounced [1, 41]. However, we found that ethnic matches avoid speaking if the language in question is an oppressed language. As one of the participants (RW51) mentioned, some health professionals, despite being able to speak Kurdish, avoided speaking, because of political implications and the devalued status of Kurdish. New studies also show that Kurdish is gradually being less spoken in the public sphere outside the family [18, 19].

Another main strategy to deal with language barriers is the provision of interpretation services. Despite its shortcomings, such as information loss during interpretation, and not being able to share private information with the interpreter [42], it has been a commonly used technique to deal with language barriers [2]. This is again a service that the Kurdish population is deprived of, because of political oppression. Consequently, the Kurdish population who are not provided HC services in their language; are not provided interpretation services; and also who are not able to easily find an ethnic match, are left to their own resources to cope with language barriers in accessing healthcare.

We found that, for our participants, social resources were almost the only resource in accessing healthcare. These social resources were being converted into cultural resources sometimes in the form of health knowledge; sometimes in the form of language skills for interpretation; other times, to economic resources, in case of a financial need to seek healthcare outside the region. These types of contributions of social resources are seen in other populations having similar problems [43]. However, the prominence of utilizing social resources in accessing healthcare in our study is mainly due to their inequitable access to economic and cultural resources which is also rooted in political dynamics. Similar to other populations having language barriers [43], the main social resource the participants mentioned was family members and neighbors rather than collective engaged communities. As such, they are dependent on their weak and fragile resources. Therefore, they can easily lose their resources which significantly interrupts their access to healthcare.

A concrete example of losing social resources is the case of referral to or preferences for (because of dissatisfaction with local HC services) cross-city HC services. This is also related to political dynamics and inequitable distribution of resources and services. The Southeast region of Turkey, which is predominantly Kurdish, has the poorest healthcare infrastructure in the country. For instance, it has the lowest number of nurses, general practitioners, and specialists [3, 44]. Inequitably distributed health equipment/infrastructure, and shortage of healthcare staff and resources have been found in other oppressed populations as well [43]. As a result, people in these regions are regularly referred to other provinces for further investigation of their health conditions and their treatments. This makes people leave their close neighborhoods where their social resources are concentrated and leads to further despair while they search for better health outcomes.

### Strengths and limitations

This is the first study that examines how and in what ways first-language affects access to health services for the Kurds in Turkey. Given that the Kurds have been politically oppressed for almost a century, it is an important population that can reveal the effects of oppression on access to healthcare. One of the strengths of the study is also using a patient-centered theoretical framework that assesses both individual and structural dimensions of accessing healthcare.

There are also some limitations of our study. The first one is about sampling. We interviewed a sample of Kurdish-speaking-only people in a geographically determined region. Therefore, our findings may not reflect the experiences of the Kurdish people living in other parts of Turkey. Moreover, although we used a maximum variation strategy in sampling, we reached some participants using snowballing method, therefore it might reflect the opinions of a group sharing similar socioeconomic and political backgrounds. Also, the contribution of women in this study was relatively lower because we couldn’t find a Kurdish-speaking female researcher to interview women. As such, the younger female participants were less communicative during the interviews. Given that gender gender modifies the effect of ethnicity for health behaviors [45], and the rate of illiteracy (consequently not speaking the Turkish) is 1.8 times higher among women than that of men in the Eastern region of Turkey [46], they are probably those who need the most but talked the least (inverse care law). Therefore, conducting further research with a higher contribution of women might add different dimensions (such as intersectionality) to this subject. The second important limitation might be related to interpretation of data. Given that oppression can be in other forms than language or ethnicity (such as race, gender, sexual orientation, socioeconomic status etc.) [29], some findings from this study might be due to other forms of oppression. For instance, we do not know about the experience of Turkish-speaking Kurds in the healthcare system; or the experience of Turks who have similar socioeconomic status as our sample. However, we are convinced that this study provides adequate evidence regarding ethno-linguistic oppression being an independent barrier in accessing healthcare. Therefore, this study point that future research may focus on the other forms of oppression in accessing healthcare. Another prospect for future studies can be conducting a similar study with younger generations who speak Turkish fluently to see ethnicity related health accessibility problems beyond language. Because despite speaking Turkish very well, racial discrimination and otherization regarding Kurdish ethnic background may sustain in a way that speaking Turkish may not buffer the accessibility barriers.

## Conclusions

First-language related barriers in accessing healthcare for ethnic/linguistic populations go beyond miscommunication and it is complicated in long-lasting political oppression and its internalization. To improve access to healthcare for such populations, at the system level, a human rights based policy shift is needed to restructure the governance and institutional systems. One of the first steps in achieving this is official recognition of oppresed populations and acknowledgement of the determinants of their health. Recognition of these populations and legalization of dealing with their problems will pave the way for more research which is usually a gap in resolving their problems. With this recognition, apart from universal strategies, multisectoral policies specifically targeting these populations should be implemented [47]. Along with this policy shift, all first-language-related suppression policies should be lifted. Particularly in places with a dominant ethnic/linguistic population, first languages should be included in all official capacities, especially in healthcare and education. In other places professional interpretation services should be provided. Similarly, the quality of local healthcare should be improved, and policies aiming to increase the number of health professionals who speak the local language should be implemented. Also, a fair distribution of economic, social and political power should be aimed to increase community empowerment. In this sense, development of a political atmosphere that enables self-empowerment of oppressed populations is critical. In this matter, civil society, Medical and Public Health associations can play a crucial advocacy role in bringing bottom-up political pressure on politicians and policy makers to take action [48].

## Supplementary Information

**Additional file 1.**

## Data Availability

Not applicable.

## Abbreviation

HC: Healthcare
