# Supplementary material for: Oppression and internalized oppression as an emerging theme in accessing healthcare: findings from a qualitative study assessing first-language related barriers among the Kurds in Turkey
Source: Int J Equity Health. 2023 Jan 7;22:6. doi: 10.1186/s12939-022-01824-z (PMC9824985; doi:10.1186/s12939-022-01824-z)
Supplement: Supplementary file 1 — Additional file 1. [file 12939_2022_1824_MOESM1_ESM.docx]

**Abstract in Kurdish**

**Zordestî û binhişkirina bindestiyê wek babetek nû di xwegihandina xizmetên tenduristiyê de: encamên lêkolîneka çawayetiyê li ser astengiyên Kurd li Tirkiyê ji ber zimanê xwe dibînin**

**Paşxane:** Di lîteratûrê de hatiye eşkere kirin ku ziman diyarkerekê sereke yê xwegihandina xizmetên tenduristiyê ye. Di piraniya lîteratûrê de, ziman wek amûrekê peywendiya di navbera nexweş û karmendên tenduristiyê de hatiye minaqeşe kirin. Lêbelê girîng e ku tesîra dîrokê û rewşa siyasî û dînamîkên hêzê li ser kiryarên mirovan bên zelal kirin. Lîteratûra di vî warî de kêm e. Di vê lêkolînê de armanca me ew bû ku em fêm bikin ka ziman çawa tesîrê li xwegihandina xizmetên tenduristiyê dike û zordestiya li ser zimanî çawa vê têkiliyê diguhore.

**Rêbaz:** Me ev lêkolîna çawayetiyê li ser serpêhatiyên nexweşên Kurd yên li Tirkiyeyê dijîn kir ku civakeka ji sed salan zêdetir e bindest e. Me di navbera salên 2018 û 2019-ê de li bajarê Şirnexê 12 hevpeyivînên kûr (hemû ji neteweya Kurd, Tirkî-neaxêv) bi stratejiya guherbariya-herî-zêde pêk anî. Me modela Levesque ya “gihîştina xizmetên tenduristiyê ji çavê nexweşan” bikar anî. Ev model hem pîvanên takekesî hem yên binesaziyê li xwe dihewîne.

**Encam:** Me dît ku dema Kurdên Tirkî-neaxêv li Tirkiyê xwe digihînin xizmetên tenduristiyê rastî gelek astengiyên girêdayê zimanî tên. Çend astengiyên me dîtîn ev bûn: xwe-negihandina zanyariyên tenduristiyê, têkiliyên lawaz di navbera nexweş û karmendên tenduristiyê de, paşvedana pêdiviyên tenduristiyê, girêdana bi kesên dî ve di dema xwegihandina xizmetên tenduristiyê de, pabendnebûna bi tedawiyan, ji xizmetan nerazîbûn û neşiyana parastina mafên tenduristiyê. Wekî encameka neasayî, me derêxist ku di bin zordestiyê de û bi rêya binhişkirina bindestiyê, prosesên astengiyên zimanî aloz dibin. Binhişkirina bindestiyê hesteka qelsbûn û dudiliyê çêdike û dihêle nexweş nexwazin xwe bigihînin xizmetên tenduristiyê; herwiha hêza wan ya takekesî û kolektîf kêm dike û têkoşîna wan bo guhortina rewşê lawaz dike.

**Pêşniyar:** Pêdivî bi veguhortineka bingehî ya siyasetê li ser bingehê mafên mirovan, û bihêzkirina civakê heye. Di asta sîstemê de, naskirina gelên bindest bi awayek fermî, naskirina pirsgirêkên wan ên tenduristiyê û diyarkerên tenduristiya wan; û tevlêkirina zimanê wan di saziyên fermî de (bi taybetî perwerde û tenduristî) pir girîng e. Dema gorankarî di vî warî de bên kirin divê dezgehên peywendîdar bên hişyarkirin ku bindestî û binhişkirina bindestiyê astengiyek giring e û divê neyê piştguhkirin. Herwiha, jiber ku zordestî ne tenê di warê ziman û neteweperestiyê de tê kirin, divê awayên dî yên bindestiyê jî di lêkolînên siberojê de bên vekolîn.
